# Advanced modeling of bioenergetics in the mitochondrial electron transport chain with emphasis on complex IV

**DOI:** 10.1002/alz.090369

**Published:** 2025-01-03

**Authors:** Marzieh Eini Keleshteri, Chris D Cadonic, Taravat Ghafourian, Ella Thomson, Wanda M Snow, Danielle McAllister, Jelena Djordjevic, Subir K Roy Chowdhury, Paul Fernyhough, Jason D Fiege, Stephanie D Portet, Benedict C Albensi

**Affiliations:** ^1^ University of Manitoba, Winnipeg, MB Canada; ^2^ St. Boniface Hospital Research, Winnipeg, MB Canada; ^3^ Department of Pharmaceutical Sciences, College of Pharmacy, Nova Southeastern University, Fort Lauderdale, FL USA

## Abstract

**Background:**

Mitochondrial bioenergetics are essential for cellular function, specifically the intricacies of the electron transport chain (ETC), with Complex IV playing a crucial role in unraveling the mechanisms governing energy production. Mathematical models offer a valuable approach to simulate these complex processes, providing insights into normal mitochondrial function and aberrations associated with various diseases, including neurodegenerative disorders. Our research focuses on introducing and refining a mathematical model, emphasizing Complex IV in the ETC, with objectives including incorporating mitochondrial activity modulation using inhibiting and uncoupling reagents, akin to oxygen consumption experiments. Rigorous validation, calibrating against Oroboros Oxygraph‐2k data from C57BL/6 mouse mitochondria, ensures accurate reproduction of dynamic bioenergetic activities. The developed graphical user interface (GUI) complements objectives, providing an in silico platform for seamless hypothesis testing (in MATLAB).

**Method:**

Employing an innovative kinetic methodology, our research integrates inhibiting reagents (oligomycin, rotenone, antimycin A, FCCP) into the developed computational model to simulate bioenergetic responses across varied physiological conditions. Optimization of the Mean Square Error (MSE) objective function using multiple optimizing algorithms, including the genetic algorithm, and calibration against Oroboros Oxygraph‐2k data using freshly isolated mitochondria from C57BL/6 mice ensures rigorous validation of the model’s precision under both unperturbed and perturbed scenarios. These outcomes unequivocally affirm the model’s efficacy in accurately simulating the intricate contributions of Complex IV to bioenergetics.

**Result:**

The outcomes highlight the model’s efficacy in reproducing bioenergetic activities, mirroring experimental outcomes. The GUI facilitates user‐friendly in silico simulations, offering a valuable complement to traditional experiments. Beyond bioenergetics, the model proves beneficial in studying mitochondrial dysfunction, presenting insights into neurodegenerative diseases. The model’s potential for early detection and therapeutic intervention contributes to advancements in understanding and treating neurological disorders.

**Conclusion:**

Our refined mathematical model successfully simulates mitochondrial bioenergetics, emphasizing Complex IV dynamics. Validated against experimental data, the model accurately reproduces bioenergetic activities and demonstrates the potential for studying mitochondrial dysfunction and neurodegenerative diseases. The integration of inhibiting and uncoupling reagents, along with the user‐friendly GUI, enhances accessibility and usability. Our research contributes to advancing the medical understanding, emphasizing the role of computational models in unraveling mitochondrial complexities in neurological disorders.

